# Impact mechanism of inclusive leadership on employees’ workplace wellbeing in service enterprises: a chain mediation model of relational identification and job embeddedness

**DOI:** 10.3389/fpsyg.2025.1679002

**Published:** 2025-10-15

**Authors:** Zhuquan Yang

**Affiliations:** School of Tourism and Sport Health, Hezhou University, Hezhou, China

**Keywords:** inclusive leadership, workplace wellbeing, chain mediation, social identity theory, service enterprises, gender moderation

## Abstract

In service enterprises, employees’ workplace wellbeing is not only crucial for individual performance and mental health, but also directly affects organizational stability and customer satisfaction. Drawing on social identity theory (SIT), this study constructs and tests a chain mediation model in which inclusive leadership influences employees’ workplace wellbeing through relational identification and job embeddedness. Based on a questionnaire survey of 386 frontline employees in service enterprises in South China, the study employs structural equation modeling (SEM) and bootstrapping analysis. The results show that: (1) inclusive leadership has a significant positive effect on employees’ workplace wellbeing; (2) relational identification and job embeddedness do not exert significant mediation effects when considered independently; (3) relational identification enhances workplace wellbeing significantly through increased job embeddedness, forming a valid chain mediation path; and (4) gender plays a moderating role in some paths, with male employees relying more on structural embeddedness for wellbeing and female employees being more influenced by emotional identification. This study reveals the underlying psychological mechanism by which inclusive leadership enhances workplace wellbeing, enriching the literature on leadership and employee psychological wellbeing, and providing practical guidance for optimizing employee management strategies in service enterprises.

## 1 Introduction

In today’s increasingly service-oriented economic environment, employee workplace wellbeing has become a critical factor for the sustainable development of organizations (Roberson and Perry, 2022; Tran and Carey, 2017). This is particularly true in service enterprises, where employees are constantly exposed to customer interactions, high emotional labor demands, and job mobility risks (McGinley et al., 2019). Their psychological health and workplace wellbeing not only affect individual performance and organizational cohesion, but also determine customer satisfaction and the stability of brand image (Correia Leal et al., 2023; Wu et al., 2024). Therefore, how to effectively enhance employees’ workplace wellbeing through internal management mechanisms-especially leadership behavior-has emerged as an important topic in the fields of organizational behavior and human resource management (Baquero, 2023; Riches et al., 2024).

In recent years, inclusive leadership-a leadership style characterized by respect, acceptance, empowerment, and psychological safety-has garnered widespread attention in both organizational practice and academic research (Tran and Carey, 2017; Umrani et al., 2024). Inclusive leaders actively listen to employees’ voices, encourage diverse expressions, and respect individual differences, which helps foster high-quality leader-member relationships and enhances employees’ sense of belonging, self-worth, and responsibility (Ashmawy, 2023; Roberson and Perry, 2022). Existing studies have shown that inclusive leadership positively influences employees’ organizational commitment, innovative behavior, and psychological engagement (Bao et al., 2025; Shafaei et al., 2024; Tran and Carey, 2017). However, the internal mechanism through which it affects employees’ workplace wellbeing has yet to be systematically explored.

To gain a deeper understanding of this mechanism, this study adopts social identity theory (SIT) as its core theoretical foundation. According to this theory, individuals in organizations are not merely performers of task roles; they also construct their social self through identification with leaders, teams, and the organization itself (Tajfel and Turner, 2004). Leadership behavior plays a crucial role in shaping employees’ identity-particularly inclusive leadership, which fosters a fair, respectful, and open interaction climate (Umrani et al., 2024). Such an environment enhances employees’ relational identification, defined as the sense of self-worth and belonging derived from their relationship with the leader (Sluss and Ashforth, 2007; Zhong et al., 2024).

Furthermore, SIT suggests that once individuals develop a sense of identification, they are more inclined to pursue emotional attachment and behavioral engagement within the organization, which manifests as higher levels of job embeddedness (Wang, 2024; William Lee et al., 2014). Job embeddedness reflects the degree of connection between employees and their organization, encompassing social links, organizational fit, and perceived sacrifice upon leaving (Kiazad et al., 2015). The progression from relational identification to job embeddedness forms a critical psychological pathway for cultivating employee loyalty and workplace wellbeing (Mitchell et al., 2001). In the context of service enterprises, this psychological transition from identification to embeddedness is especially salient-employees often develop deeper organizational attachment after establishing relational identification with an inclusive leader, thereby enhancing their workplace wellbeing (Cai et al., 2022; Lu et al., 2016).

A review of the literature reveals that existing studies have primarily examined the positive effects of inclusive leadership on outcome variables such as employee innovation, organizational commitment, and work engagement (Bao et al., 2025; Choi et al., 2015; Randel et al., 2018; Yasin et al., 2023). However, few have systematically explored the impact pathways of inclusive leadership from the perspective of employee workplace wellbeing, and even fewer have considered the chain-mediating role of relational identification and job embeddedness in the psychological construction process. This suggests a potential intrinsic connection between inclusive leadership and employee workplace wellbeing. Therefore, it is necessary to draw upon SIT to investigate the chain-mediating mechanism and path logic involving relational identification and job embeddedness, in order to fill this gap in the literature. This study aims to provide theoretical insights and practical implications for service enterprises seeking to optimize leadership practices, enhance employee wellbeing, and improve organizational stability.

## 2 Theoretical foundation and research hypotheses

### 2.1 Social identity theory

Social identity theory, proposed by Turner and Oakes (1986), emphasizes that individuals do not define themselves solely based on personal characteristics, but rather through their social relationships with others or groups-forming a self-concept of “who we are (Tajfel and Turner, 2004).” In organizational contexts, employee behavior and psychological states are not merely determined by task characteristics, but are deeply influenced by their sense of social identification with the organization, team, and especially the leader (Ashforth and Mael, 1989). Once individuals develop identification with a significant other or group, they are more likely to exhibit a high level of responsibility, loyalty, and proactive behavior.

In this study, SIT provides a powerful explanatory framework for understanding how inclusive leadership influences employee workplace wellbeing through psychological mechanisms. First, inclusive leadership, characterized by openness, active listening, and acceptance, fosters an atmosphere of respect, trust, and fairness. This enables employees to feel understood and supported in their relationships with leaders, thereby strengthening their relational identification (Sluss and Ashforth, 2007). As a component of one’s “social self,” relational identification enhances employees’ sense of belonging and meaning, and makes them more willing to pursue long-term psychological and behavioral engagement within the organization.

Second, relational identification serves as an emotional foundation through which employees identify with both the leader and the organization, further promoting their level of job embeddedness. Job embeddedness reflects the extent of connection and fit between employees and the organization, comprising three dimensions: social links, value fit, and perceived sacrifice (Mitchell et al., 2001). Driven by social identification, employees are more likely to resonate with organizational goals, form emotional attachment, maintain behavioral stability, and sustain role continuity, thereby enhancing their embeddedness (Steffens et al., 2017).

Finally, employees who are embedded in both emotional identification and organizational connection are more likely to perceive satisfaction, achievement, and fulfillment from their work-manifesting as higher levels of workplace wellbeing (Karanika-Murray et al., 2015). Therefore, SIT not only reveals the potential link between inclusive leadership and employee wellbeing, but also provides strong theoretical support for the construction of a chain-mediating pathway through relational identification and job embeddedness.

### 2.2 Research hypotheses

#### 2.2.1 The relationship between inclusive leadership and workplace wellbeing

In today’s high-pressure, high-interaction service-oriented organizational environments, employees’ emotional states and wellbeing have increasingly become key factors influencing their work engagement, service quality, and organizational stability (Brunetto et al., 2014; Kundi et al., 2022). Workplace wellbeing, as a comprehensive reflection of employees’ subjective occupational experience, encompasses dimensions such as positive emotional experiences related to work, life satisfaction, and a sense of meaning at work (Litchfield, 2021). Enhancing employees’ workplace wellbeing not only strengthens their sense of organizational belonging and loyalty, but also helps reduce turnover rates and improve organizational performance (Anand et al., 2024).

Leadership style is a critical organizational context factor that affects employees’ psychological states (Kyambade et al., 2024). Among various styles, inclusive leadership-which emphasizes respect, listening, and empowerment-creates a psychologically safe, expressive, and diversity-embracing environment within the organization (Ashmawy, 2023). This leadership style has been shown to significantly foster employees’ positive psychological responses and emotional value experiences (Carmeli et al., 2010; Randel et al., 2018). Specifically, inclusive leadership promotes employees’ perceived value and sense of respect in the workplace by validating their opinions and emotions, encouraging their participation in decision-making, and providing developmental support, thereby enhancing their overall happiness at work (Javed et al., 2019).

From the perspective of SIT, inclusive leadership strengthens employees’ positive construction of their relationships with leaders, teams, and the organization, helping to form a positive “social self” concept (Ashforth and Mael, 1989). This identification encourages employees to see themselves as part of the organization, which leads to stronger workplace wellbeing in an environment of recognition, support, and acceptance (Sluss and Ashforth, 2007).

Empirical research also supports a significant positive relationship between inclusive leadership and employees’ psychological health and subjective wellbeing (Hanh Tran and Choi, 2019; Rogozińska-Pawełczyk, 2023). For example, Choi et al. (2017) found that inclusive leadership enhances employees’ psychological safety and sense of meaning, which in turn promotes more positive evaluations of their work and emotional feedback. Kim et al. (2024) similarly found that in high emotional labor settings, inclusive leadership effectively buffers employee stress and promotes higher levels of wellbeing. Therefore, this study proposes the following hypothesis:

H1: Inclusive leadership has a significant positive impact on employees’ workplace wellbeing.

#### 2.2.2 The mediating role of relational identification

First, inclusive leadership has a significantly positive effect on employees’ relational identification. Relational identification refers to individuals’ self-concept formed through interactions with important others, particularly emphasizing emotional bonds and role identification with specific individuals such as leaders (Sluss and Ashforth, 2007). Inclusive leadership, by demonstrating respect, active listening, and acceptance, allows employees to feel trusted and valued, thereby strengthening their positive evaluation of and sense of belonging with their leaders (Rogozińska-Pawełczyk, 2023). As a result, they are more likely to incorporate the relationship with their leader into their self-identity (Ashforth et al., 2008). According to SIT, leaders’ behaviors serve as important social cues that influence employees’ understanding of their roles and relationships within the organization (Alhashedi et al., 2021). Hence, in an inclusive climate, employees are more likely to derive positive social identity from the “I–leader relationship,” forming a stronger sense of relational identification (Guo et al., 2022).

Second, relational identification can significantly enhance employees’ workplace wellbeing. Relational identification not only reinforces employees’ sense of belonging and identity within the organization, but also enhances their sense of purpose at work and emotional connection, thereby increasing their wellbeing (Salin et al., 2023). When employees feel recognized and trusted in their relationship with the leader, they are more likely to experience positive emotions such as satisfaction, pride, and belonging, which improves their subjective evaluation of work (Akkan and Guzman, 2022). Furthermore, strong relational identification can heighten employees’ alignment with organizational goals, leading to greater meaning and emotional engagement in their work, which ultimately contributes to improved workplace wellbeing on both cognitive and affective levels (Ashforth et al., 2008).

Based on these two arguments, relational identification is likely to mediate the relationship between inclusive leadership and employee workplace wellbeing. Social identity theory emphasizes that individuals construct self-identity through social relationships, which in turn influence their emotional and behavioral responses. Through positive interactions, inclusive leadership fosters employees’ relational identification, thereby promoting emotional positivity and identity value, and ultimately enhancing their workplace wellbeing (Baquero, 2023). This process reflects an emotional transmission pathway that begins with external leadership behavior, flows through internal psychological identification, and results in subjective wellbeing. Existing research supports this pathway. For instance, Bao et al. (2025) found that under supportive leadership, employees who identified strongly with their leaders tended to exhibit more positive psychological states and behaviors. Therefore, this study proposes the following hypothesis:

H2: Relational identification mediates the relationship between inclusive leadership and employees’ workplace wellbeing.

#### 2.2.3 The mediating role of job embeddedness

First, inclusive leadership can effectively enhance employees’ job embeddedness. Job embeddedness refers to the extent to which employees are connected to their job and organization, including the degree of fit, links, and perceived sacrifice associated with leaving the organization (Mitchell et al., 2001). Inclusive leadership fosters a work atmosphere that is safe, stable, and respectful by actively listening to employees, providing developmental opportunities, and offering emotional support (Roberson and Perry, 2022). This leadership style helps employees build stronger emotional bonds and identification with the organization (Umrani et al., 2024). Particularly in service-oriented enterprises, such leadership encourages employees to align with organizational culture and values, increasing their sense of fit and willingness to stay, thus improving their level of job embeddedness (Yasin et al., 2023).

Second, job embeddedness has a significant positive impact on employees’ workplace wellbeing. Employees with high levels of job embeddedness often experience a stronger sense of emotional belonging, tighter social networks, and greater role stability-factors that help reduce job uncertainty and turnover anxiety, while enhancing emotional stability and wellbeing (Ampofo and Karatepe, 2022). Studies have shown that when employees possess more social links, cognitive fit, and perceived costs of leaving the organization, they are more likely to derive satisfaction, security, and a sense of meaning from their work, thereby reporting higher levels of subjective workplace wellbeing (Ahmad et al., 2023). In addition, job embeddedness can also boost intrinsic motivation, enabling employees to experience sustained emotional positivity through active engagement at work (Ramaite et al., 2022).

Based on this logic, job embeddedness may serve as a mediator between inclusive leadership and workplace wellbeing. From the perspective of SIT, inclusive leadership strengthens employees’ social self by encouraging identification with the organization and alignment with its values. This drives employees to embed themselves more deeply into the organizational structure and culture (Ashforth et al., 2008; Tajfel and Turner, 2004). Such embeddedness is not only reflected in objective structures such as networks and job fit, but also manifests as emotional extension of belonging and self-fulfillment-representing a behavioral expression of social identity at the structural level (Wang, 2024). Therefore, inclusive leadership may indirectly enhance workplace wellbeing by promoting higher levels of job embeddedness. Accordingly, this study proposes the following hypothesis:

H3: Job embeddedness mediates the relationship between inclusive leadership and employees’ workplace wellbeing.

#### 2.2.4 The chain-mediating role of relational identification and job embeddedness

In organizational contexts, employees’ relational identification not only shapes their emotional sense of belonging but may also strengthen their structural connection with the organization-namely, their job embeddedness (Ahmad et al., 2023). Relational identification refers to the self-concept that employees construct based on interactions with significant others (e.g., leaders), and represents a specific manifestation of social identity within interpersonal relationships (Sluss and Ashforth, 2007). In contrast, job embeddedness reflects the multi-dimensional connection between employees and their organization, including social links, value fit, and perceived sacrifice (Mitchell et al., 2001).

From the perspective of SIT, as employees develop identification with their leaders, this relationship gradually becomes internalized as part of their social identity (Ashforth and Mael, 1989; Hogg, 2016). This identity then expands to encompass broader organizational contexts, triggering deeper psychological alignment and behavioral embeddedness (Wang, 2024). Once employees emotionally identify with their leader, this positive affective state motivates them to seek meaning and presence within the organization, increases their willingness to collaborate with teams, assimilate into organizational culture, and uphold organizational goals-ultimately resulting in a higher degree of structural job embeddedness (Dursun and Karadaş, 2025).

Moreover, prior research suggests that the psychological safety and identity clarity derived from relational identification can foster more stable social links and role fit, while enhancing employees’ perception of the costs associated with leaving the organization-corresponding to the three core dimensions of job embeddedness (Lo Presti et al., 2024). In other words, relational identification is not only an affective-cognitive outcome but also a crucial psychological precursor for motivating employees’ willingness to become embedded within the organization (Hu et al., 2025).

Therefore, based on the logic of SIT, relational identification may serve as a bridging variable that facilitates employees’ psychological progression from emotional identification to structural connection. This pathway constitutes a key mechanism through which inclusive leadership enhances workplace wellbeing. Accordingly, this study proposes the following hypothesis:

H4: Relational identification, by enhancing job embeddedness, plays a chain-mediating role between inclusive leadership and employees’ workplace wellbeing.

#### 2.2.5 The moderating role of gender

Gender, as a key individual characteristic, has been widely recognized as a significant moderator in the field of organizational behavior (Aftab et al., 2020). Prior studies have demonstrated systematic differences between male and female employees in terms of cognitive style, emotional responsiveness, relational orientation, and organizational commitment, which may shape how leadership styles influence employee outcomes (Collins et al., 2014). Specifically: Female employees tend to be more sensitive to emotional support and interpersonal relationships (Verma et al., 2018). Male employees are often more responsive to structural resources such as job embeddedness, due to a greater tendency toward rational analysis and organizational fit (Afsar and Badir, 2016). Moreover, gender differences may influence how inclusive leadership impacts employees’ wellbeing and their sense of belonging in the workplace (Caminong et al., 2023).

Based on these theoretical and empirical foundations, we propose the following integrated moderation hypothesis:

H5: Gender moderates the effects of inclusive leadership on relational identification, job embeddedness, and workplace wellbeing across all relevant pathways.

**FIGURE 1:**
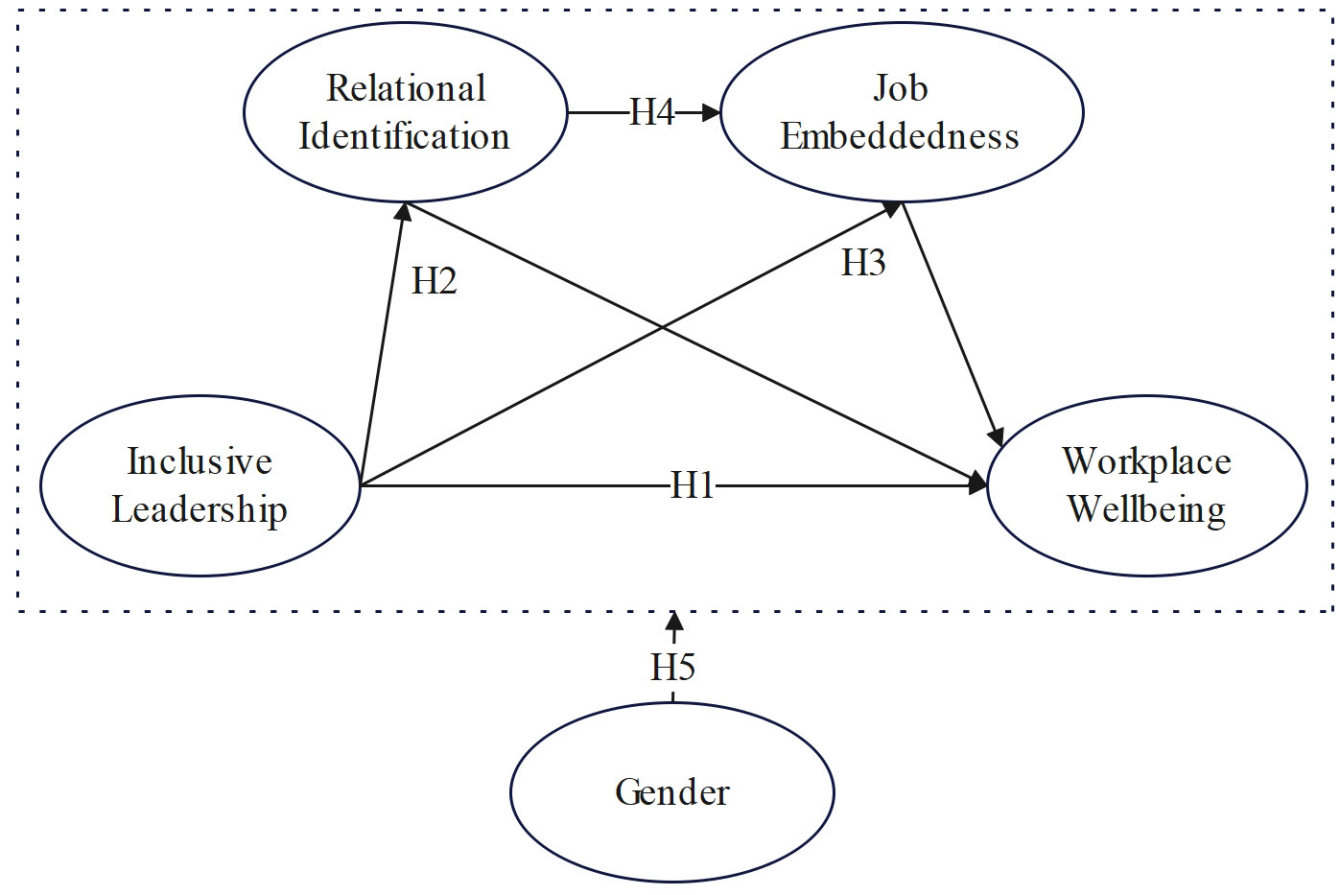
Conceptual model. IL, inclusive leadership; JE, job embeddedness; RI, relational identification; WW, workplace wellbeing.

Based on the above hypotheses, the conceptual model for this study is presented in Figure 1.

## 3 Research design

### 3.1 Variable definitions and measurement scales

This study focuses on four core variables: inclusive leadership, relational identification, job embeddedness, and workplace wellbeing. All items were measured using a five-point Likert scale (1 = strongly disagree, 5 = strongly agree). Established and internationally validated instruments were adopted and linguistically adapted to fit the Chinese context. The definitions and sources of each variable are as follows:

#### 3.1.1 Inclusive leadership

Inclusive leadership refers to the behavioral characteristics of leaders who demonstrate openness, respect, acceptance, and encourage employee participation in the management process. A 9-item scale developed by Carmeli et al. (2010) was used, which covers three dimensions: Openness, Availability, and Accessibility. This scale has been widely applied in organizational behavior research and has shown good reliability and validity. Example items include: My supervisor encourages me to express new ideas, My supervisor is willing to discuss work-related issues with me.

#### 3.1.2 Relational identification

Relational identification refers to the self-concept that employees construct through their interactions with significant others (e.g., direct supervisors), representing the degree to which they internalize and value these relationships. A 6-item scale developed by Walumbwa and Hartnell (2011) was adopted, which is specifically designed to measure subordinate-supervisor relationships in organizational settings. Example items include: I consider my relationship with my supervisor as part of my self-identity, I feel that my relationship with my supervisor is meaningful.

#### 3.1.3 Job embeddedness

Job embeddedness reflects the extent to which employees are connected to and aligned with their organization, as well as their reasons for staying. This study adopted the short-form Job Embeddedness Scale developed by Crossley et al. (2007), which consists of 7 items and covers the three core dimensions of Links, Fit, and Sacrifice. Example items include: I have many good interpersonal relationships at work, If I left my current job, I would lose a lot of valuable things.

#### 3.1.4 Workplace wellbeing

Workplace wellbeing was measured using the Eudaimonic Workplace Well-being Scale developed by Bartels et al. (2019). This scale distinguishes between two dimensions: interpersonal wellbeing (e.g., connectedness with others at work) and intrapersonal wellbeing (e.g., personal growth, meaning, and emotional vitality). The scale includes 8 items in total. Example items include: I feel emotionally energized at work, I believe my work is meaningful, I feel a sense of camaraderie with my colleagues.

### 3.2 Sample selection and data collection

#### 3.2.1 Research subjects

The participants of this study were frontline employees from several large-scale service enterprises in South China, including resorts, hotels, and cultural tourism groups. Employees in these organizations typically face high levels of customer pressure and emotional labor demands. Given the significant impact of leadership styles on their psychological states and work experiences, they serve as appropriate empirical samples for this study (Etikan et al., 2016).

#### 3.2.2 Data collection procedures

Data were collected through anonymous questionnaires via two main channels:

Offline surveys: Paper-based questionnaires were distributed on-site in collaboration with the HR departments of participating organizations. Employees completed the surveys voluntarily and returned them collectively.

Online surveys: An electronic version of the questionnaire was distributed via the Wenjuanxing platform and shared in organizational chat groups. Respondents were required to confirm their voluntary participation and ensure independent responses.

Before the formal survey, the research team conducted pilot interviews and a pretest of the scales with a subset of employees to ensure clarity and logical consistency of the questionnaire items. Minor revisions were made based on feedback. All participants were informed that the survey was entirely voluntary, the data would be used exclusively for academic purposes, and personal information would be kept strictly confidential, with no relevance to any performance evaluation or assessment.

To enhance data quality, the research team provided real-time Q&A support through online platforms during the survey period, addressing any issues or misunderstandings encountered by respondents. This approach helped ensure the accuracy and completeness of the responses (Golzar et al., 2022).

#### 3.2.3 Sample control and data screening

Several quality control measures were implemented to improve the validity of the data: Participants were explicitly informed of the academic purpose of the study and the confidentiality of their responses. Reverse-coded and consistency-check items were included to identify invalid responses. Responses with unusually short completion times (e.g., under 90 s) or highly repetitive answer patterns were excluded.

A total of 400 questionnaires were distributed. After removing invalid responses, 386 valid questionnaires were retained, yielding a response rate of 96.5%, which meets the recommended sample size requirements for structural equation modeling (SEM) analysis (Bentler, 1990; Loehlin, 2004).

## 4 Data analysis and research results

### 4.1 Descriptive statistics

To better understand the demographic characteristics of the respondents, this study conducted a frequency analysis of 386 valid questionnaires, focusing on gender, age, education level, and years of work experience. The results are as follows:

The detailed demographic distribution of the respondents is shown in Table 1.

**TABLE 1:** Sample demographic information.

Item	Category	Frequency	Percentage (%)	Cumulative percentage (%)
Gender	Male	172	44.56	44.56
	Female	214	55.44	100
Age	18–25	75	19.43	19.43
	26–30	64	16.58	36.01
	31–40	119	30.83	66.84
	41–50	100	25.91	92.75
	51–60	26	6.74	99.48
	Above 60	2	0.52	100
Edu	Junior high school and below	111	28.76	28.76
	Senior high school	94	24.35	53.11
	Junior college	102	26.42	79.53
	Bachelor’s degree	73	18.91	98.45
	Master’s degree and above	6	1.55	100
Work year	Below 5	178	46.11	46.11
	5–10	100	25.91	72.02
	11–15	62	16.06	88.08
	16–20	26	6.74	94.82
	Above 20	20	5.18	100
		386	100	100

Gender: Among the respondents, 172 were male (44.56%) and 214 were female (55.44%). The proportion of female employees was slightly higher than that of male employees, reflecting the relatively high participation of women in frontline positions within the service industry.

Age: The majority of participants were aged 31–40 (119 respondents, 30.83%), followed by those aged 41–50 (100 respondents, 25.91%), 18–25 (75 respondents, 19.43%), and 26–30 (64 respondents, 16.58%). Only 32 respondents (8.29%) were aged 51 and above. These figures indicate that the sample was primarily composed of young and middle-aged employees, consistent with the active labor force characteristics and youth-oriented employment trends of the service industry.

Educational Background: Over half of the participants held a high school diploma or below. Specifically, 111 respondents (28.76%) had completed junior high school or less, and 94 (24.35%) had a high school or vocational school diploma. A total of 96 respondents (24.87%) had an associate degree, and 73 (18.91%) held a bachelor’s degree. Only 6 respondents (1.55%) had a postgraduate degree or higher. This distribution reflects the generally modest educational attainment levels of frontline employees in China’s service sector.

Years of Work Experience: Employees with 5 years or less of work experience constituted the largest proportion (178 respondents, 46.11%), suggesting relatively high labor mobility and a younger workforce. Those with 6–10 years of experience accounted for 100 respondents (25.91%), while 11–15 years (62 respondents, 16.06%) and 16 years or more (46 respondents, 11.92%) were less represented. This indicates that overall tenure is relatively short, and organizational accumulation is low, emphasizing the need to strengthen retention through management practices.

In summary, the demographic structure of the sample reflects the actual human resource profile of frontline workers in service-oriented enterprises. The data exhibit good representativeness and provide a solid foundation for subsequent empirical analyses.

### 4.2 Common method bias test

Since all variables in this study were measured using self-reported questionnaires collected from a single source at a single time point, the potential influence of common method bias (CMB) was assessed using Harman’s one-factor test. This method involves conducting an unrotated exploratory factor analysis on all measurement items to determine whether a single factor accounts for the majority of the variance (Harman, 1976).

The results revealed six factors with eigenvalues greater than 1, collectively explaining 88.189% of the total variance. The first factor accounted for 29.802% of the variance, which is well below the critical threshold of 40%, suggesting that common method bias is not a significant concern in this study (Podsakoff et al., 2003).

In addition, the Kaiser-Meyer-Olkin (KMO) value was 0.976, and Bartlett’s test of sphericity was significant (χ^2^ = 21212.471, df = 561, *p* < 0.001), indicating sufficient inter-item correlations and the suitability of the data for factor analysis.

Taken together, these results suggest that common method bias is not a serious issue in this study, allowing for subsequent structural modeling analysis.

### 4.3 Reliability and validity analysis

To ensure the quality of the measurement instruments, this study conducted a Confirmatory Factor Analysis (CFA) to assess the internal consistency reliability, convergent validity, and discriminant validity of all constructs. The reliability and validity results are summarized in Table 2.

**TABLE 2:** Reliability and validity test of the scale.

Factor	Observed variable	Coef.	Std. Error	z	Std. Estimate	Cronbach	C.R	AVE
Openness	Openness 1	1	–	–	0.939	0.961	0.961	0.896
	Openness 2	0.976	0.023	42.974	0.97			
	Openness 3	0.91	0.025	35.929	0.931			
Availability	Availability 1	1	–	–	0.935	0.965	0.966	0.875
	Availability 2	0.999	0.031	32.347	0.907			
	Availability 3	1.066	0.027	39.271	0.953			
	Availability 4	1.155	0.03	38.141	0.947			
Accessibility	Accessibility 1	1	–	–	0.962	0.951	0.951	0.907
	Accessibility 2	0.994	0.024	42.202	0.943			
Relational identification	Relational identification 3	1	–	–	0.818	0.963	0.965	0.737
	Relational identification 10	1.101	0.05	22.118	0.886			
	Relational identification 1	1.051	0.059	17.896	0.725			
	Relational identification 2	1.002	0.052	19.411	0.816			
	Relational identification 4	1.092	0.048	22.807	0.903			
	Relational identification 6	1.042	0.045	23.059	0.909			
	Relational identification 8	1.096	0.062	17.581	0.758			
	Relational identification 5	1.033	0.044	23.413	0.916			
	Relational identification 7	1.063	0.045	23.809	0.926			
	Relational identification 9	0.923	0.04	22.812	0.902			
Job embeddedness	Job embeddedness 1	1	–	–	0.908	0.968	0.969	0.816
	Job embeddedness 2	1.136	0.044	26.035	0.866			
	Job embeddedness 3	0.998	0.031	32.383	0.926			
	Job embeddedness 5	1.235	0.046	27.111	0.883			
	Job embeddedness 7	1.156	0.04	29.215	0.906			
	Job Embeddedness 4	1.127	0.035	32.542	0.94			
	Job embeddedness 6	1.167	0.046	25.16	0.891			
Interpersonal workplace wellbeing	Interpersonal dimenshion 1	1	–	–	0.945	0.975	0.975	0.908
	Interpersonal dimenshion 2	1.019	0.023	43.963	0.965			
	Interpersonal dimenshion 3	1.052	0.026	41.201	0.951			
	Interpersonal dimenshion 4	1.036	0.026	40.354	0.949			
Intrapersonal workplace wellbeing	Intrapersonal dimension 1	1	–	–	0.945	0.973	0.973	0.901
	Intrapersonal dimension 2	0.993	0.023	43.458	0.963			
	Intrapersonal dimension 3	1.049	0.025	41.725	0.956			
	Intrapersonal dimension 4	0.936	0.025	37.197	0.932			

#### 4.3.1 Internal consistency reliability

Cronbach’s alpha coefficients and Composite Reliability (CR) were used to evaluate the internal consistency of the measurement scales. All constructs reported Cronbach’s α values ranging from 0.951 to 0.975, significantly exceeding the recommended threshold of 0.70, indicating excellent internal reliability. Specifically:

For Inclusive Leadership, the three dimensions showed high reliability: Openness (α = 0.961), Availability (α = 0.965), and Accessibility (α = 0.951);

Relational Identification (α = 0.963), Job Embeddedness (α = 0.968), and Workplace Well-being (Interpersonal α = 0.975; Intrapersonal α = 0.973) also demonstrated excellent internal consistency;

All constructs exhibited Composite Reliability (CR) values above 0.90, further confirming the robustness and stability of the measurement model.

#### 4.3.2 Convergent validity

Convergent validity was assessed using standardized factor loadings and the Average Variance Extracted (AVE). The results revealed that:

All factor loadings exceeded 0.70, with most ranging between 0.85 and 0.95, indicating strong item representation of their respective constructs;

Average Variance Extracted values for all constructs were above 0.70 (minimum = 0.737; maximum = 0.908), far surpassing the recommended threshold of 0.50, confirming satisfactory convergent validity (Fornell and Larcker, 1981).

#### 4.3.3 Discriminant validity

Discriminant validity was assessed using the Fornell-Larcker criterion by comparing the square root of each construct’s AVE with its correlations with other constructs (Fornell and Larcker, 1981). As shown in the correlation matrix (see Table 3):

**TABLE 3:** Discriminant validity test of latent variables.

Variable	Openness	Availability	Accessibility	Relational identification	Job embeddedness	Interpersona workplace wellbeing	Intrapersonal workplace wellbeing
Openness	0.947						
Availability	0.929	0.936					
Accessibility	0.893	0.936	0.952				
Relational identification	0.805	0.824	0.841	0.859			
Job embeddedness	0.752	0.729	0.761	0.86	0.903		
Interpersonal workplace wellbeing	0.712	0.718	0.732	0.8	0.814	0.953	
Intrapersonal workplace wellbeing	0.775	0.775	0.769	0.822	0.878	0.876	0.949

The square root of Relational Identification’s AVE was 0.859, which was greater than its correlations with Openness (*r* = 0.805), Availability (*r* = 0.824), and other constructs;

Job Embeddedness showed a square root of AVE of 0.903, also significantly higher than its correlations with all other constructs.

These findings indicate that each latent variable is empirically distinct from the others, confirming satisfactory discriminant validity of the measurement model.

### 4.4 Model fit evaluation

To assess the adequacy of the proposed structural equation model with respect to the observed data, several commonly recommended model fit indices were evaluated using AMOS. These included χ^2^/df, GFI, TLI, CFI, RMSEA, and SRMR. The results are summarized as follows (see Table 4):

**TABLE 4:** Model fit test.

Common indicators	χ 2	df	*p*	χ 2/df	GFI	RMSEA	RMR	CFI	NFI	NNFI
Evaluation criteria	–	–	>0.05	<3	>0.9	<0.10	<0.05	>0.9	>0.9	>0.9
Value	1431.656	477	0	3.001	0.823	0.072	0.027	0.955	0.935	0.948
Other indicators	TLI	AGFI	IFI	PGFI	PNFI	PCFI	SRMR	RMSEA 90% CI		
Evaluation criteria	>0.9	>0.9	>0.9	>0.5	>0.5	>0.5	<0.1	–		
Value	0.948	0.779	0.956	0.66	0.795	0.812	0.03	0.068 ∼ 0.076		

The chi-square statistic was χ^2^ = 1431.656 with 477 degrees of freedom, yielding a χ^2^/df = 3.001. Although slightly above the ideal threshold of 3, this value is still considered acceptable given the relatively large sample size (*N* = 386), as chi-square is sensitive to sample size (Bollen, 1989);

The Goodness-of-Fit Index (GFI = 0.823) and Adjusted Goodness-of-Fit Index (AGFI = 0.779) were close to the recommended threshold of 0.90, suggesting an acceptable model fit;

Incremental fit indices all exceeded the recommended value of 0.90, indicating excellent fit: Tucker–Lewis Index (TLI = 0.948), Comparative Fit Index (CFI = 0.955), Normed Fit Index (NFI = 0.935), and Non-Normed Fit Index (NNFI = 0.948);

The Root Mean Square Error of Approximation (RMSEA = 0.072) with a 90% confidence interval of [0.068, 0.076] was below the acceptable threshold of 0.08; both Root Mean Square Residual (RMR = 0.027) and Standardized Root Mean Square Residual (SRMR = 0.030) were well below the cutoff of 0.05, indicating small residuals and controlled model error.

In summary, all fit indices reached acceptable or good levels, demonstrating that the proposed structural model has satisfactory overall fit to the data and is appropriate for further analysis of path coefficients and mediation effects.

### 4.5 Hypothesis testing

#### 4.5.1 Direct effect testing

To examine the direct relationships among the key variables, this study employed SEM to estimate path coefficients while controlling for demographic variables such as gender, age, education level, and years of work experience. The results are as follows (see Table 5):

**TABLE 5:** Model test.

Variable	RI					JE					WW					WW				
	B	Std. Error	*t*	*p*	β	B	Std. Error	*t*	*p*	β	B	Std. Error	t	p	β	B	Std. Error	t	p	β
Constant	0.406*	0.167	2.436	0.015	–	0.253	0.159	1.586	0.113	–	1.142**	0.166	6.893	0	–	0.808**	0.121	6.7	0	–
Gender	−0.045	0.047	−0.961	0.337	−0.026	−0.068	0.044	−1.535	0.126	−0.039	−0.025	0.047	−0.539	0.59	−0.017	0.031	0.034	0.915	0.361	0.021
Age	−0.04	0.025	−1.613	0.107	−0.057	0.061**	0.023	2.602	0.01	0.086	0.036	0.024	1.485	0.138	0.06	0.029	0.018	1.611	0.108	0.048
Edu	0.014	0.024	0.58	0.562	0.019	0.01	0.023	0.464	0.643	0.014	0.004	0.024	0.187	0.852	0.007	−0.008	0.017	−0.458	0.647	−0.012
Work year	0.023	0.023	0.993	0.321	0.032	0.025	0.022	1.153	0.249	0.034	0	0.023	−0.013	0.99	0	−0.024	0.017	−1.467	0.143	−0.038
IL	0.878**	0.029	30.615	0	0.845	0.130*	0.051	2.563	0.011	0.122	0.715**	0.029	25.07	0	0.789	0.189**	0.039	4.896	0	0.208
RI						0.776**	0.049	15.929	0	0.757						0.166**	0.047	3.488	0.001	0.19
JE																0.470**	0.039	12.121	0	0.552
R^2^	0.715					0.756					0.629					0.809				
Adjust R^2^	0.711					0.752					0.624					0.805				
*F*-value	F (5,380) = 190.533, *p* = 0.000					F (6,379) = 195.913, *p* = 0.000					F (5,380) = 129.014, *p* = 0.000					F (7,378) = 228.451, *p* = 0.000				

*Indicates *p* < 0.05;

**Indicates *p* < 0.01;

***Indicates *p* < 0.001.

Inclusive Leadership significantly and positively influences Workplace Wellbeing (β = 0.208, *t* = 4.896, *p* < 0.001), thus supporting Hypothesis H1;

Inclusive Leadership has a significantly positive effect on Relational Identification (β = 0.845, *t* = 30.615, *p* < 0.001), supporting the first half of Hypothesis H2;

Inclusive Leadership also exhibits a significant direct effect on Job Embeddedness (β = 0.122, *t* = 2.563, *p* < 0.05), indicating that even after controlling for Relational Identification, a direct pathway still exists;

Relational Identification significantly predicts Job Embeddedness (β = 0.757, *t* = 15.929, *p* < 0.001), further confirming the motivating role of relational identity in the downstream mechanism;

Both Relational Identification (β = 0.166, *t* = 3.488, *p* < 0.01) and Job Embeddedness (β = 0.552, *t* = 12.121, *p* < 0.001) have significant and positive effects on Workplace Wellbeing, lending support to the second halves of Hypotheses H2 and H3, respectively.

These results indicate that the proposed structural paths are largely significant and that the model demonstrates strong structural validity.

#### 4.5.2 Mediating effect testing

To further examine the mediating mechanisms of Relational Identification and Job Embeddedness in the relationship between Inclusive Leadership and Workplace Wellbeing, this study employed the bootstrapping method (5,000 resamples, 95% confidence interval) (Freedman, 1981). The results are as follows (see Tables 6, 7):

**TABLE 6:** Analysis of effect processes.

Effect	Item	Effect	SE	*t*	p	LLCI	ULCI
Direct effect	IL→WW	0.189	0.039	4.896	0	0.113	0.264
	IL→RI	0.878	0.029	30.615	0	0.822	0.934
	IL→JE	0.13	0.051	2.563	0.011	0.031	0.229
Indirect effect process	RI→JE	0.776	0.049	15.929	0	0.68	0.871
	RI→WW	0.166	0.047	3.488	0.001	0.072	0.259
	JE→WW	0.47	0.039	12.121	0	0.394	0.546
Total effect	IL→WW	0.715	0.029	25.07	0	0.659	0.771

**TABLE 7:** Mediation effect test.

Item	Effect	Boot SE	BootLLCI	BootULCI	z	p
IL→RI→WW	0.145	0.083	−0.005	0.323	1.751	0.08
IL→JE→WW	0.061	0.035	−0.007	0.139	1.742	0.081
IL→RI→JE→WW	0.32	0.078	0.207	0.51	4.109	0

The indirect effect of Inclusive Leadership→Relational Identification→Workplace Wellbeing was 0.145, with a 95% confidence interval of [−0.005, 0.323], which includes zero and is therefore not statistically significant, suggesting that this single mediation path is unstable;

The indirect effect of Inclusive Leadership→Job Embeddedness→Workplace Wellbeing was 0.061, with a 95% confidence interval of [−0.007, 0.139], which is also not significant, indicating that Job Embeddedness alone does not serve as a significant mediator;

However, the chain mediation path of Inclusive Leadership→Relational Identification→Job Embeddedness→ Workplace Wellbeing yielded a significant indirect effect of 0.320, with a 95% confidence interval of [0.207, 0.510], which does not include zero (*z* = 4.109, *p* < 0.001), indicating a robust and statistically significant mediation.

In summary, Relational Identification and Job Embeddedness jointly form a significant chain mediating mechanism between Inclusive Leadership and Workplace Wellbeing. In contrast, neither variable demonstrates a significant mediating effect on its own. These findings suggest that the formation of employees’ wellbeing is more dependent on a sequential psychological construction path from identification to embeddedness, thereby supporting Hypothesis H4.

#### 4.5.3 Moderating effect of gender

To examine the moderating effect of gender on the path from Inclusive Leadership→Relational Identification→Job Embeddedness→Workplace Wellbeing, this study conducted a Multi-Group Structural Equation Modeling (MGSEM) analysis to assess the significance of gender differences (Kock, 2014). Based on the gender variable, the sample was divided into two groups: male employees (*n* = 172) and female employees (*n* = 214), and the following steps were applied to test whether specific paths differed significantly between the two groups.

First, a baseline model was estimated with all path parameters freely estimated across groups. Then, for each target path, a constrained model was constructed by setting the parameter to be equal across the two groups. By comparing the chi-square difference (Δχ^2^) between the constrained and baseline models, the significance of the gender-based moderation was assessed.

The results showed that several paths differed significantly between gender groups (as shown in Tables 8, 9):

**TABLE 8:** Test of gender moderation effects.

Model	Δχ^2^	P	Δdf
**Basic model**				
Constrained model: Inclusive leadership→Relational identification	8.412	0.004***	1.000
Constrained model: Relational identification→Workplace wellbeing	17.940	0.000***	1.000
Constrained model: Inclusive leadership→Job embeddedness	1.102	0.294	1.000
Constrained model: Relational identification→Job embeddedness	0.022	0.881	1.000
Constrained model: Job embeddedness→Workplace wellbeing	21.189	0.000***	1.000
Constrained model: Inclusive leadership→Workplace wellbeing	6.699	0.010*	1.000

*Indicates *p* < 0.05;

**Indicates *p* < 0.01;

***Indicates *p* < 0.001.

**TABLE 9:** Comparison for moderating effects of gender.

Path	Male		Female	
	Std. coefficients	*t*-value	Std. coefficients	*t*-value
Inclusive leadership→Relational identification	0.912***	19.923	0.878***	19.923
Relational identification→Workplace wellbeing	−0.179	−1.672	0.427***	4.637
Inclusive leadership→Job embeddedness	−0.105	−0.911	0.035	0.439
Relational identification→Job embeddedness	0.959***	7.557	0.85***	9.242
Job embeddedness→Workplace wellbeing	0.828***	11.572	0.438***	6.269
Inclusive leadership→Workplace wellbeing	0.336***	4.126	0.100	1.559

*Indicates *p* < 0.05;

**Indicates *p* < 0.01;

***Indicates *p* < 0.001.

For the path Inclusive Leadership→Relational Identification, Δχ^2^ = 8.412, *p* = 0.004, indicating a significant difference. The path coefficient was stronger for male employees (β = 0.912, *t* = 19.923) than for female employees (β = 0.878, *t* = 19.923).

For the path Relational Identification→Workplace Wellbeing, Δχ^2^ = 17.940, *p* < 0.001, indicating that female employees were more sensitive to relational identification in enhancing wellbeing (female β = 0.427, *t* = 4.637; male β = −0.179, *t* = −1.672).

For the path Job Embeddedness→Workplace Wellbeing, Δχ^2^ = 21.189, *p* < 0.001, the path coefficient was significantly higher for male employees (β = 0.828, *t* = 11.572) than for female employees (β = 0.438, *t* = 6.269).

Additionally, the path Inclusive Leadership→Workplace Wellbeing also showed a significant gender difference (Δχ^2^ = 6.699, *p* = 0.010), with males (β = 0.336) reporting a stronger direct effect than females (β = 0.100).

By contrast, the paths Inclusive Leadership→Job Embeddedness (Δχ^2^ = 1.102, *p* > 0.05) and Relational Identification→Job Embeddedness (Δχ^2^ = 0.022, *p* > 0.05) did not differ significantly between gender groups.

In summary, gender played a significant moderating role in several paths of the model: Male employees were more likely to develop strong relational identification when perceiving inclusive leadership; Job Embeddedness had a greater impact on Workplace Wellbeing among males; Female employees, on the other hand, were more influenced by Relational Identification in enhancing their sense of wellbeing. These findings suggest that employees of different genders respond differently to leadership behaviors and psychological mechanisms. Therefore, gender-sensitive and differentiated management strategies should be adopted in practical human resource and leadership practices.

## 5 Conclusion and discussion

### 5.1 Research conclusion

Grounded in SIT, this study empirically examined the relationships among Inclusive Leadership, Relational Identification, Job Embeddedness, and Employees’ Workplace Wellbeing. A chain mediation model was developed and tested, with gender introduced as a moderating variable for multi-group comparison. Based on SEM and bootstrapping analysis of survey data from 386 frontline employees in service-oriented enterprises, the following key findings emerged:

Inclusive leadership has a significant positive effect on employees’ workplace wellbeing, confirming prior research emphasizing leadership behavior as a crucial driver of employees’ subjective wellbeing (Bartels et al., 2019; Carmeli et al., 2010; Choi et al., 2017; Javed et al., 2019; Kim et al., 2024). By fostering a climate of respect, voice, and empowerment, inclusive leaders provide employees with psychological safety and value affirmation, thereby stimulating emotional positivity and job satisfaction, which ultimately enhances overall wellbeing (Roberson and Perry, 2022; Tran and Carey, 2017; Umrani et al., 2024).

The individual mediating effects of relational identification and job embeddedness were not significant. While inclusive leadership significantly predicted both relational identification and job embeddedness, neither variable independently mediated the relationship between inclusive leadership and wellbeing. This suggests that although inclusive leadership can strengthen employees’ relational bonds and structural ties to the organization, these effects may not suffice to boost wellbeing unless they are integrated into a more cohesive psychological mechanism. This finding echoes Sluss and Ashforth (2007) view that relational identification serves as an initial step in social identity construction, and aligns with Mitchell et al. (2001) assertion that the effectiveness of job embeddedness depends on broader contextual support (Ashforth et al., 2008; Lo Presti et al., 2024; Wang, 2024).

The chain mediation pathway Inclusive Leadership→ Relational Identification→Job Embeddedness→Workplace Wellbeing was found to be significant, representing a core contribution of this study. This implies that employees’ wellbeing is not directly influenced by leadership behavior alone, but is progressively shaped through a psychological process of “first identifying, then embedding.” This result is consistent with the fundamental proposition of SIT: that individual motivation and emotional experience are shaped by the ongoing construction and negotiation of self-identity within social structures (Hogg, 2016; Steffens et al., 2017; Tajfel and Turner, 2004).

Gender plays a significant moderating role in specific paths. Male employees demonstrated greater sensitivity to inclusive leadership in forming relational identification, suggesting that they are more responsive to the relational cues from inclusive leaders. In contrast, female employees showed a stronger relationship between relational identification and wellbeing, indicating a higher dependence on emotional-relational cues. This aligns with proposition that gender moderates emotional responses and identity mechanisms in organizational settings (Akkan and Guzman, 2022; Guo et al., 2022).

In conclusion, this study not only validates the positive influence of inclusive leadership on employees’ workplace wellbeing, but also uncovers the underlying “affective–structural–psychological” chain mechanism through which this influence occurs. The moderating role of gender further enriches our understanding of how different employee groups perceive and respond to leadership behaviors. These findings offer both a systematic theoretical framework and practical guidance for enhancing wellbeing in service-oriented organizational contexts (Anand et al., 2024; Baquero, 2023; Cai et al., 2022).

### 5.2 Practical implications

From the perspective of organizational management practices, this study uncovers the psychological mechanism by which inclusive leadership enhances employees’ workplace wellbeing through relational identification and job embeddedness, while also identifying the moderating role of gender. These findings offer the following actionable insights for service-oriented enterprises seeking to optimize leadership styles, enhance employee wellbeing, and stabilize their workforce:

Develop an inclusive leadership system to strengthen employees’ emotional identification. The results show that inclusive leadership behaviors-such as active listening, emotional support, and empowerment-significantly promote relational identification, which in turn enhances employees’ wellbeing and sense of belonging. Therefore, organizations should implement leadership training programs, establish behavioral guidelines, and design performance evaluation systems that reinforce inclusive management principles. Special emphasis should be placed on frontline service contexts, where cultivating a respectful, diverse, and trust-based work environment is crucial (Bao et al., 2025; Sluss and Ashforth, 2007; Umrani et al., 2024).

Leverage the “identification–embeddedness” mechanism to strengthen organizational attachment. The study reveals that emotional support alone is insufficient for enhancing wellbeing; employees must also develop stable structural ties with the organization. Enterprises should therefore move from relational management to embeddedness management by institutional means. Practical strategies include: optimizing job rotation systems to improve person–job fit, developing internal social platforms to foster peer connections, and introducing incentive mechanisms that enhance perceived loss upon exit-thus deepening both emotional and structural attachment to the organization (Kiazad et al., 2015; Lo Presti et al., 2024; Wang, 2024).

Implement gender-sensitive management by recognizing differential psychological responses. Multi-group SEM results indicate that male employees are more likely to convert relational identification into strong embeddedness, which contributes to higher levels of wellbeing. Female employees, by contrast, exhibit greater sensitivity to relational cues, deriving wellbeing more directly from relational satisfaction. These differences call for tailored management strategies: for female employees, organizations might focus on emotional incentives and frequent interpersonal feedback; for male employees, emphasis could be placed on structured career development paths and role stability to support sustained embeddedness (Akkan and Guzman, 2022; Choi et al., 2017; Hu et al., 2025).

Integrate employee wellbeing into the organization’s sustainable development strategy. In service industries, employees’ wellbeing is closely tied to customer satisfaction, serving as a key driver of long-term performance and brand loyalty. Organizations should elevate wellbeing from a peripheral concern in “welfare management” to a strategic priority. This includes establishing employee wellbeing metrics, incorporating emotional commitment and organizational identification into key performance indicators (KPIs), and embedding “people-centered” values into corporate culture to build a high-quality employee experience system (Anand et al., 2024; Riches et al., 2024; Salin et al., 2023).

### 5.3 Theoretical contribution

First, the study advances the theoretical understanding of inclusive leadership by uncovering its indirect pathway to workplace wellbeing through relational identification and job embeddedness. While prior research has primarily explored the direct effects of inclusive leadership (Bao et al., 2022; Choi et al., 2017), our findings reveal that inclusive leadership facilitates employees’ wellbeing not merely by offering support, but by shaping employees’ self-concept (via identification) and reinforcing their organizational ties (via embeddedness). This enriches the SIT framework by demonstrating how leadership behaviors are internalized through both emotional and structural mechanisms.

Second, this study contributes to the contingent perspective of leadership effectiveness by incorporating gender as a moderator across six key relational and structural paths. By highlighting gender-specific differences in how inclusive leadership translates into wellbeing-e.g., males showing stronger identification, females responding more affectively-our findings support and extend gendered leadership theories (Caminong et al., 2023), suggesting that leadership outcomes are not uniform but shaped by employees’ socialized orientations and cognitive-affective processing patterns.

### 5.4 Limitations and future research

Although this study constructed and validated a relatively comprehensive model exploring the mechanisms by which inclusive leadership, relational identification, and job embeddedness influence employee wellbeing, and preliminarily revealed how organizational emotional resources enhance psychological wellbeing, several limitations remain and warrant further investigation:

Cross-sectional design limits causal inference. This study employed a cross-sectional survey approach to collect data at a single time point. Although structural equation modeling and mediation analysis were used to enhance analytical rigor, it is difficult to fully eliminate common method bias and the possibility of reverse causality. Future research should consider adopting longitudinal designs or experimental approaches to better support the dynamic causal chain of “inclusive leadership→identification and embeddedness→wellbeing.”

Sample representativeness is limited. The sample primarily consisted of frontline employees in service-oriented industries, with certain biases in industry and regional distribution. This may limit the generalizability of the findings. Future studies should expand the sampling scope to include diverse sectors such as manufacturing, IT, education, and healthcare, and examine the heterogeneity across age groups and tenure levels in the psychological mechanisms underlying employee wellbeing.

The variable framework does not fully capture the multidimensional nature of wellbeing. While this study considered key psychological constructs such as leadership style, identification, and embeddedness, it did not incorporate other important factors known to influence wellbeing, such as psychological safety, meaningfulness of work, and positive affect. Future research could integrate insights from positive organizational behavior to develop more complex structural models that reveal multi-level and multi-path mechanisms of wellbeing formation.

## Data Availability

The original contributions presented in this study are included in this article/supplementary material, further inquiries can be directed to the corresponding author.
